# Isolated Facial Vein Thrombophlebitis Caused by *Fusobacterium nucleatum*: A Lemierre-Variant Case

**DOI:** 10.1155/crdi/9938125

**Published:** 2025-08-15

**Authors:** Nicole Oska, Deni Peterson, Kendall Brothers, Ragamayi Maramraju, Asem Ayyad, Brianna Hohmann

**Affiliations:** ^1^School of Medicine, Wayne State University, Detroit, Michigan, USA; ^2^Department of Internal Medicine, Henry Ford Hospital, Detroit, Michigan, USA

## Abstract

**Background:** Lemierre's syndrome is an uncommon yet potentially fatal infection, classically secondary to bacterial pharyngeal infections. It is typically characterized by bacteremia, most frequently due to *Fusobacterium necrophorum* and internal jugular vein thrombophlebitis. If untreated, septic embolization may result, potentially damaging the lungs, liver, brain, or other organs. This report describes a variant of Lemierre's syndrome in a young woman with streptococcal pharyngitis, who developed *Fusobacterium nucleatum* bacteremia and isolated facial vein thrombophlebitis, highlighting the importance of early diagnosis and treatment.

**Case Presentation:** A 33-year-old woman with no significant past medical history presented with sore throat, chills, and right-sided facial and neck pain. Initially diagnosed with streptococcal pharyngitis at an urgent care clinic, she presented to the emergency department soon afterwards due to the development of severe rigors and fever. Blood cultures revealed growth of *Fusobacterium nucleatum*, raising suspicion for Lemierre's syndrome in the setting of worsening facial and neck pain despite negative initial imaging of the soft tissue of the neck. A subsequent CT venogram confirmed isolated thrombosis of the right facial vein. The patient was treated and discharged in stable condition on a 4 week course of metronidazole and 2 weeks of ceftriaxone.

**Conclusion:** This case emphasizes the importance of maintaining suspicion for Lemierre's syndrome in patients with bacterial pharyngitis, especially when atypical symptoms such as facial pain occur. Although the internal jugular vein is most commonly affected, facial vein thrombosis may also occur. Early antibiotic treatment is critical for preventing severe complications including septic shock and embolization.

## 1. Introduction

Lemierre's syndrome is a rare infection that occurs secondary to pharyngeal infections, typically manifesting with bacteremia, internal jugular vein thrombophlebitis, and septic emboli [1]. The most common causative organism is *Fusobacterium necrophorum*, an anaerobic bacterium that is part of the oropharyngeal flora, which can extend into the parapharyngeal space leading to inflammation and thrombophlebitis of the internal jugular vein [2]. Untreated septic emboli can disperse into various sites such as the liver, brain, joints, and more commonly, the lungs [3].

We describe an atypical case of Lemierre's syndrome in a young woman with confirmed streptococcus pharyngitis who presented with sudden chills and persistent facial pain, leading to the discovery of isolated facial vein thrombophlebitis associated with *F. nucleatum* bacteremia. Our case emphasizes the importance of maintaining a high degree of suspicion for this disease given its high mortality if untreated.

## 2. Case

**Figure 1:**
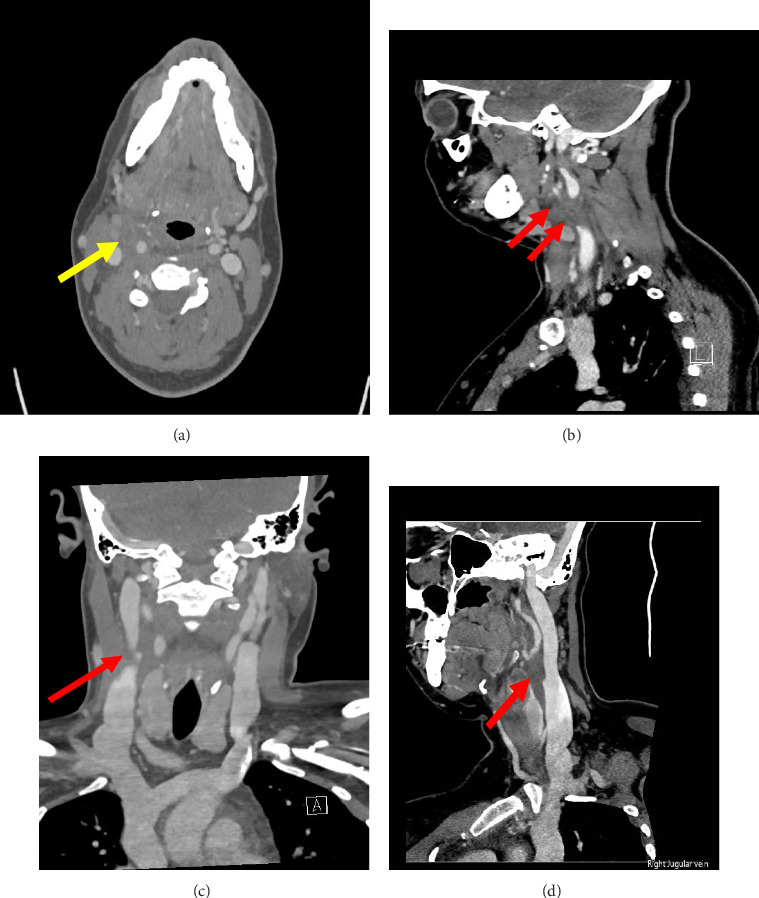
(a) Axial CT venogram revealing a small fluid collection (yellow arrow), suggestive of tonsillar abscess. (b, c, d) Coronal, sagittal, and single right internal jugular curve views demonstrating a filling defect of the right facial vein near the inflow of the right internal jugular vein (red arrows), suggestive of thrombosis.

A 33-year-old woman with no significant medical history was admitted following a 2 day history of sore throat. Prior to admission, she had presented to an urgent care office where a rapid strep test was positive, and the urgent care prescribed her azithromycin due to her penicillin allergy. That same day, she developed severe rigors and abdominal pain, prompting her presentation to the emergency department. There, she was found to be febrile and tachycardic; thus, blood cultures were drawn, and IV clindamycin was started for suspected streptococcal bacteremia given her sudden chills and abdominal pain. On admission, vitals were significant for tachycardia to 115 beats per minute, but she was no longer febrile. Laboratory studies were significant for leukopenia (2.9 K/μL) with neutrophilic predominance (93%), elevated lactate (2.8 mmol/L), and elevated D-dimer (4.07 μg/mL FEU). Initial CT of the soft tissue of the neck did not reveal any peritonsillar abscesses or vascular abnormalities. The next day, IV ceftriaxone 2 g daily was started to provide better Gram-negative coverage. In addition, the patient tested positive for *Trichomonas vaginalis* on nucleic-acid testing, so she was also started on oral metronidazole at that time. Two days later, blood cultures revealed growth of *Fusobacterium nucleatum,* speciated with MALDI-TOF mass spectrometry. The finding of anaerobic oropharyngeal flora in the blood, in addition to the patient's persistent right facial tenderness and mild swelling, suggests a Lemierre-like septic thrombophlebitis. A subsequent CT venogram of the neck demonstrated segmental thrombosis of the right facial vein in addition to the development of a small tonsillar abscess, confirming the diagnosis of Lemierre's syndrome (Figure 1).

Treatment with IV ceftriaxone was continued daily, in addition to metronidazole 500 mg three times daily to improve coverage for *Fusobacterium*. The patient remained hemodynamically stable throughout the admission and upon clinical improvement was discharged home on IV ceftriaxone via a peripherally inserted central catheter for a total course of 2 weeks and oral metronidazole for a total course of 4 weeks.

## 3. Outcome and Follow-Up

On follow-up 2 weeks after discharge, the patient reported significant improvement in symptoms, but right facial tenderness was still persistent on exam. A repeat CT venogram was obtained which demonstrated resolution of the tonsillar abscess and no new acute thrombosis. The patient was instructed to continue the antibiotic regimen as originally planned.

## 4. Discussion

This case underscores the importance of having a high suspicion for Lemierre's syndrome in the setting of bacterial pharyngitis. Young patients with Lemierre's syndrome typically present with signs of pharyngeal disease and sepsis who are then found to have positive blood cultures and internal jugular venous thrombosis on imaging [1]. Older patients, in contrast, often present with more severe complications, such as abscesses [1]. Our patient presented with signs of sepsis in the hospital in the setting of streptococcal pharyngitis. Lemierre's syndrome is a relatively rare condition, but it can be life-threatening through septic embolization if it is not identified and treated quickly. Our patient had initial symptoms that were more consistent with uncomplicated streptococcal pharyngitis, but the progressive facial pain, chills, and abdominal pain indicated a more severe pathogenesis. Blood cultures that were positive for *Fusobacterium nucleatum*, a common bacterial pathogen for Lemierre's syndrome, alongside a CT venogram demonstrating venous thrombosis of the right facial vein, confirmed the diagnosis of Lemierre's syndrome. Interestingly, it is unusual to have streptococcal pharyngitis and subsequent *Fusobacterium* septic thrombophlebitis, as the original pathogen is typically the one isolated in blood cultures; to our knowledge, isolated facial vein thrombosis caused by *F. nucleatum* after documented streptococcal pharyngitis has not been previously reported. The other cases reported in the literature isolated *F. necrophorum* in blood cultures or did not report the causative pathogen [4, 5].

Current literature on Lemierre's syndrome raises questions regarding the use of anticoagulation following diagnosis. Although recommendations for anticoagulation vary in the literature [6–8], a meta-analysis on the role of anticoagulation in the treatment regimen found that anticoagulation does not appear to have a statistically significant effect on mortality [9]. Therefore, given our patient's condition was relatively mild, stable, and detected early, we made a decision to defer anticoagulation. In comparison, prompt treatment with antibiotics is known to prevent morbidity and mortality. Current recommendations favor treatment with metronidazole in combination with a beta-lactam or carbapenem because *F. necrophorum*, the most studied causative pathogen, is widely sensitive to metronidazole [1, 10]. Furthermore, case reports on *F. nucleatum* Lemierre's syndrome have supported treatment with a beta-lactam or carbapenem and metronidazole, and in vitro studies have found that *F. nucleatum* is widely sensitive to beta-lactams, carbapenems, and metronidazole [11–13]. The length of antibiotic treatment varies, with some studies recommending 3  to 5 weeks of intravenous antibiotic therapy, while in some cases, 2 weeks of intravenous antibiotic therapy and a transition to oral antibiotics is appropriate. Our patient's condition was not severe compared to other cases outlined in the literature, and she remained stable and afebrile throughout most of the admission; therefore, the decision to treat with 2 weeks of intravenous ceftriaxone and 4 weeks of oral metronidazole was appropriate for her. If antibiotics are delayed, the infection will spread hematogenously through venous circulation, leading to devastating complications like septic shock or necrotic lung lesions [1].

Isolated facial vein thrombophlebitis is considered an atypical variant of Lemierre's syndrome [8]. Current literature shows that the most commonly thrombosed vessel on imaging in patients diagnosed with Lemierre's disease is the internal jugular vein, with very few documented cases reporting isolated thrombosis of the facial vein and none due to *F. nucleatum* [10]. While neck pain is a common symptom of internal jugular vein septic thrombophlebitis, our patient had facial pain due to thrombophlebitis of the facial vein. Physicians should investigate causes of pain outside of the neck in patients presenting with pharyngitis as it could indicate thrombophlebitis in less typical locations.

This case highlights the need for vigilance in diagnosing atypical presentations of Lemierre's syndrome, as early recognition and prompt antibiotic treatment are critical to preventing severe complications.

## Data Availability

The data that support the findings of this study are available from the corresponding author upon reasonable request.
